# A PCR protocol to establish standards for routine mycoplasma testing that by design detects over ninety percent of all known mycoplasma species

**DOI:** 10.1016/j.isci.2023.106724

**Published:** 2023-04-26

**Authors:** Dominik Siegl, Marie Kruchem, Sandrine Jansky, Emma Eichler, Dorothe Thies, Udo Hartwig, Detlef Schuppan, Ernesto Bockamp

**Affiliations:** 1 Institute of Translational Immunology (TIM), University Medical Center of the Johannes Gutenberg-University, Mainz 55131, Germany; 2 Department of Medicine III Hematology & Medical Oncology, University Medical Center of the Johannes Gutenberg-University, Mainz 55131, Germany; 3 ImmuneNTech GmbH, Wendelsheim 55234, Germany; 4 Division of Gastroenterology, Beth Israel Deaconess Medical Center, Harvard Medical School, Boston, MA 02215, USA; 5 Research Center for Immunotherapy, University Medical Center of the Johannes Gutenberg-University, Mainz 55131, Germany

**Keywords:** Microbiology, Mycology, Sequence analysis

## Abstract

Mycoplasma infection leads to false and non-reproducible scientific data and poses a risk to human health. Despite strict guidelines calling for regular mycoplasma screening, there is no universal and widely established standard procedure. Here, we describe a reliable and cost-effective PCR method that establishes a universal protocol for mycoplasma testing. The applied strategy utilizes ultra-conserved eukaryotic and mycoplasma sequence primers covering by design 92% of all species in the six orders of the class *Mollicutes* within the phylum Mycoplasmatota and is applicable to mammalian and many non-mammalian cell types. This method can stratify mycoplasma screening and is suitable as a common standard for routine mycoplasma testing.

## Highlights

• Efficient PCR/qPCR procedure to detect mycoplasma infections in cell culture
• Ultra-conserved primers covering by design >90% of all Mycoplasmatota species
• Simple, generally applicable and reliable protocol for routine mycoplasma testing

## Graphical abstract

**Figure:**
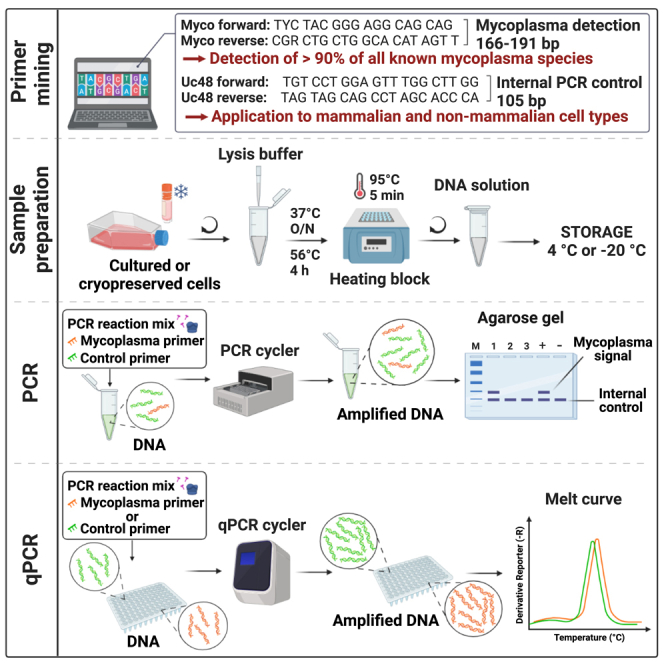

## Introduction

Mycoplasmas colonize vertebrate, fish and plant cells and belong to the phylum Mycoplasmatota and the class *Mollicutes* comprising the six different orders *Acholeplasmatales, Anaeroplasmatales, Entomoplasmatales, Haloplasmatales, Mycoplasmatales* and *Mycoplasmoidales* which from here on will be referred to as mycoplasmas.^1^ Originally derived from Gram-positive bacteria by a process of reductive evolution, mycoplasmas represent the smallest free-living organisms, lack a cell wall, and have very small genomes.^2^^,^^3^ Mycoplasmas can cause various human diseases. Among the pathogenic mycoplasma species, *Mycoplasmoides* *pneumoniae* causes atypical pneumonia and also contributes to infections of the joints, blood or the central nervous system.^4^ Another well-known pathogenic mycoplasma is *Mycoplasmoides genitalium* that can give rise to sexually transmitted infections.^4^ Besides that, mycoplasmas represent a common problem in cell culture experiments and it is generally assumed that between 10 and 35% of all cell lines are contaminated with mycoplasmas.^5^^,^^6^ Of interest, several mycoplasma species, originally derived from bovine, porcine and human cells, have completely lost their host species tropism and now colonize many different tissue culture cells.^7^ The loss of host specificity thus explains why *Acholeplasma laidlawii*, *Mycoplasmopsis arginini*, *Mycoplasmopsis fermentans*, *Mesomycoplasma hyorhinis*, and *Metamycoplasma orale* *(M. orale)* represent the most common species found in cell culture.^8^

Although for decades the main source of mycoplasmas was contaminated bovine serum, today infections mostly occur by cross-contamination.^7^ In culture, mycoplasmas can grow in the medium or remain attached to cell membranes,^9^ and some species, such as *Mycoplasmopsis fermentans,* have the ability to actively invade eukaryotic cells to colonize the cytoplasm.^6^^,^^7^ Mycoplasmas also survive cryopreservation^10^ as well as direct contact with liquid nitrogen and have been reported to spread in liquid nitrogen tanks,^10^^,^^11^ further highlighting the need for regular testing. In case initial sources of contamination go undetected, mycoplasma infection gradually spreads and is often long-term preserved in frozen aliquots. Additional unresolved obstacles to effectively combat mycoplasmas emerge from their reduced size and lack of cell walls, thus evading sterilization using standard filtration techniques and the detection by light microscopy. Equally, mycoplasmas do not produce turbidity in culture medium and are resistant to commonly applied tissue culture antibiotics.^10^

Although mycoplasma infections are often not considered as a possible source, they can have a considerable impact on tissue culture conditions and normal cell behavior as e.g. infection likely affects the growth rate, morphology and viability of cells but phenotypic changes are generally mild.^12^^,^^13^ Although in certain mycoplasma species metabolic processes are not fully understood, known mechanisms include arginine metabolism, fermentation of sugars to lactate or oxidation of pyruvate or lactate. Depending on the strain, one, two or all three of the above-mentioned pathways may be used.^14^^,^^15^ Although minor phenotypic changes in the morphology of mycoplasma infected host cells are often caused by nutrient deprivation, these alterations are not easily noticeable when growth medium is replaced regularly.^13^^,^^16^ Most importantly, mycoplasmas can interfere with normal cell physiology and signaling. Alterations in gene expression,^17^ interference with signal transduction,^18^ impairment of nucleic acid incorporation,^19^^,^^20^ induction of oxidative stress,^21^ promotion of chromosomal alterations and instability^22^ and malignant transformation,^23^ likely caused by inhibiting *TP53* tumor suppressor function,^24^ have been described.

Historically, the gold standard for mycoplasma detection has been culturing on specific mycoplasma broth or agar plates.^25^ However, this microbiological method takes a minimum of one to two weeks of incubation time and because only some mycoplasma strains grow on such substrates, many remain undetected. In addition, mycoplasmas can be visualized following staining of bacterial DNA with Hoechst dye and microscopic inspection.^26^ In our experience, Hoechst staining can provide fast readouts, but we found that interpreting the results is at times difficult. In contrast, the use of specific monoclonal antibodies against well-defined epitopes (for example^27^) allows defined mycoplasma detection, but limits the readout to one or a few species and thus does not allow detection of a broad spectrum of different mycoplasma variants. Although in recent years more high-end mycoplasma testing methods such as microchip electrophoresis, DNA microchip detection and surface-enhanced Raman spectroscopy have been developed,^28^^,^^29^^,^^30^ the most common technique used for mycoplasma screening is PCR, which allows both high sensitivity and specificity.

Currently, many laboratories use different commercial kits and in-house methods for mycoplasma detection. However, a generally accepted and inexpensive experimental standard for routine mycoplasma testing is missing. Here, we present a PCR method for routine screening of mammalian and many non-mammalian cell types that is suitable as a universal standard for mycoplasma detection.

## Results

### Target identification and primer specificity

To identify highly conserved 16S rRNA mycoplasma-specific regions, we resorted to the NCBI Bacterial 16S Ribosomal RNA RefSeq Targeted Loci Project (Accession PRJNA33175). First, we designed primer pairs based on 60 pre-selected mycoplasma strains and reanalyzed these using the total dataset that contained 25796 entries at the time of analysis. This bioinformatics approach identified one fitting primer pair for global mycoplasma detection. The mapped primers identify 272 listed database entries representing 204 different species of which 198 (97%) are on-target (mycoplasmas) and six (3%) are off-target (non-mycoplasmas). When the dataset is analyzed on strain level, the primers identify 233 different strains, of which 226 (97%) are on-target and seven (3%) are off-target. Table 1 indicates the different mycoplasma-as well as non-mycoplasma genera, their coverage (on genera and strain level), and the percentages. Table S1 provides an extended list showing the exact individual matches for each on- and off-target direct sequence match as well as potential and missing matches. In summary, the selected primer combination matches with 198 out of 216 mycoplasma species or 226 out of 246 mycoplasma strains, providing a total coverage of 92% in both cases.

**Table 1:** Genera- and strain-specific on-target and off-target matches

Genera	Coverage (matched species/all species)	Percentage species [%]	Coverage (matched strains/all strains)	Percentage strains [%]
**On-target (mycoplasma)**				

*Acholeplasma*	8/9	89	11/12	92
*Alteracholeplasma*	2/2	100	2/2	100
*Entomoplasma*	3/3	100	3/3	100
*Haploplasma*	2/3	67	2/3	67
*Malacoplasma*	1/4	25	1/4	25
*Mariniplasma*	1/1	100	1/1	100
*Mesomycoplasma*	13/13	100	16/16	100
*Mesoplasma*	11/11	100	12/12	100
*Metamycoplasma*	22/22	100	28/28	100
*Mycoplasma*	39/41	95	42/44	95
*Mycoplasmoides*	5/6	83	7/8	88
*Mycoplasmopsis*	43/44	98	53/56	95
*Paracholeplasma*	2/2	100	2/2	100
*Spiroplasma*	34/38	89	34/38	89
*Ureaplasma*	9/9	100	9/9	100
*Williamsoniioplasma*	3/3	100	3/3	100

**Off-target (non-mycoplasma)**				

*Brachyspira*	1/9	11	1/10	10
*Micrococcoides*	1/1	100	1/1	100
*Nitriliruptor*	1/1	100	1/1	100
*Oligella*	1/4	25	1/6	17
*Peptococcus*	2/2	100	3/3	100

### Functional validation of the PCR assay

**Figure 1:**
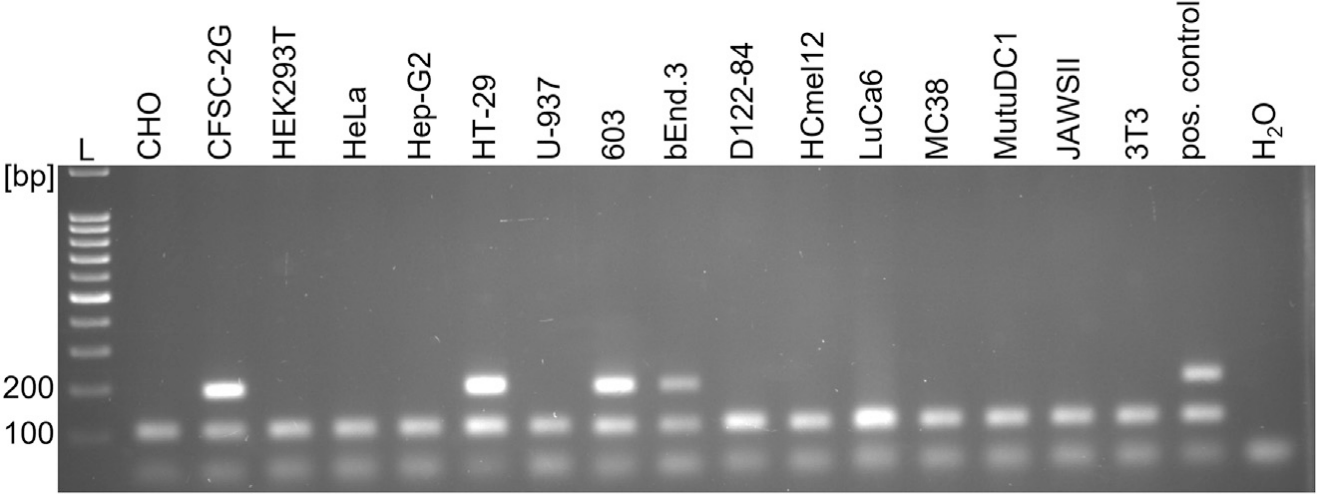
Detection of mycoplasma infection in different cell lines using PCR PCR testing was performed on the indicated human, mouse, hamster and rat cell lines. The lower 105 bp band represents the internal control PCR product and the upper band (between 166 and 191 bp) indicates the presence of mycoplasma DNA. L = DNA ladder, pos. control = extracted DNA of mycoplasma-infected cells, H_2_O = negative control.

Mycoplasmas colonize eukaryotic cell membranes and invade eukaryotic cells. To increase the likelihood for mycoplasma detection and to include an internal control, we utilized eukaryotic cell extracts for PCR amplification. As shown in Figure 1, a four-primer PCR with Myco- and Uc48-primer pairs amplified a 105 bp PCR product used as positive control in all tested cell culture samples, thus directly confirming the presence of eukaryotic DNA and indicating a productive PCR. In addition, from the 16 cell lines tested, in the reactions using DNA extracts from CFSC-2G, HT-29, 603 and bEnd.3 samples, a 166–191 bp DNA amplification product was generated, indicating the presence of mycoplasma DNA.

### Four-primer PCR sensitivity

**Figure 2:**
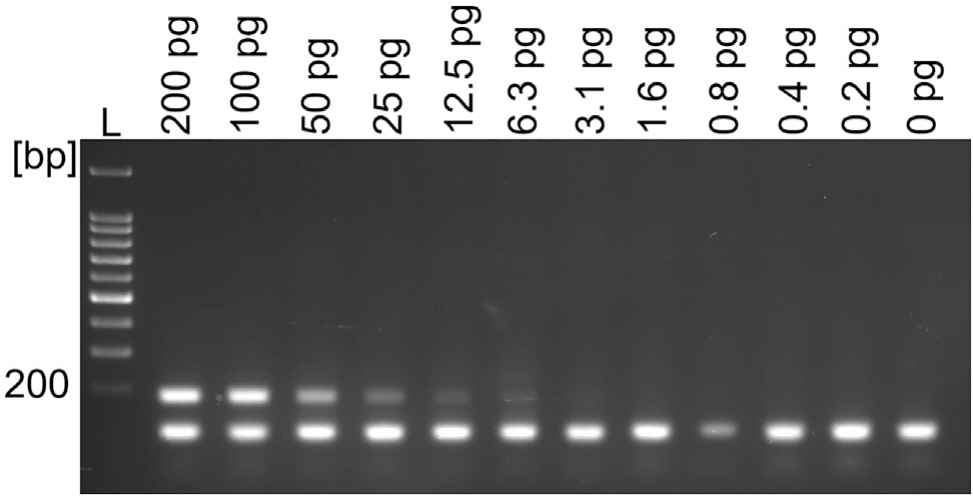
Sensitivity of the four-primer mycoplasma PCR *M. orale* DNA was serially diluted and mixed with DNA extracted from 1x10^6^ HEK293T cells. *M. orale* DNA concentrations between 200 pg and 0.2 pg were tested. L = DNA ladder.

To assess the limit of detection (LOD) for mycoplasma DNA, four-primer PCR was performed on genomic *M. orale* DNA. To reflect the presence of genomic cell DNA in the standard mycoplasma detection protocol, a constant amount of extracted HEK293T DNA was added to each mycoplasma DNA serial dilution sample. As shown in Figure 2, the detection limit for *M. orale* DNA was 6.3 pg or 8.21x10^3^ genomic copies.^31^ To demonstrate the robustness of the assay in the context of different cell lines, we mixed *M. orale* DNA with mycoplasma-free genomic DNA from twelve cell lines. As shown in Figure S1, the inclusion of 50 pg *M. orale* DNA to all tested genomic cell line samples, produced in each case the expected *M. orale* 188 bp PCR fragment.

### Mycoplasma detection using quantitative PCR

Many laboratories use quantitative PCR (qPCR) for routine PCR assays. For this reason, we subjected different cell lines to qPCR and tested for mycoplasma infection. Because mycoplasma-specific and control PCR amplification products are very similar in size, we performed parallel reactions using each primer set in a separate reaction.

**Figure 3:**
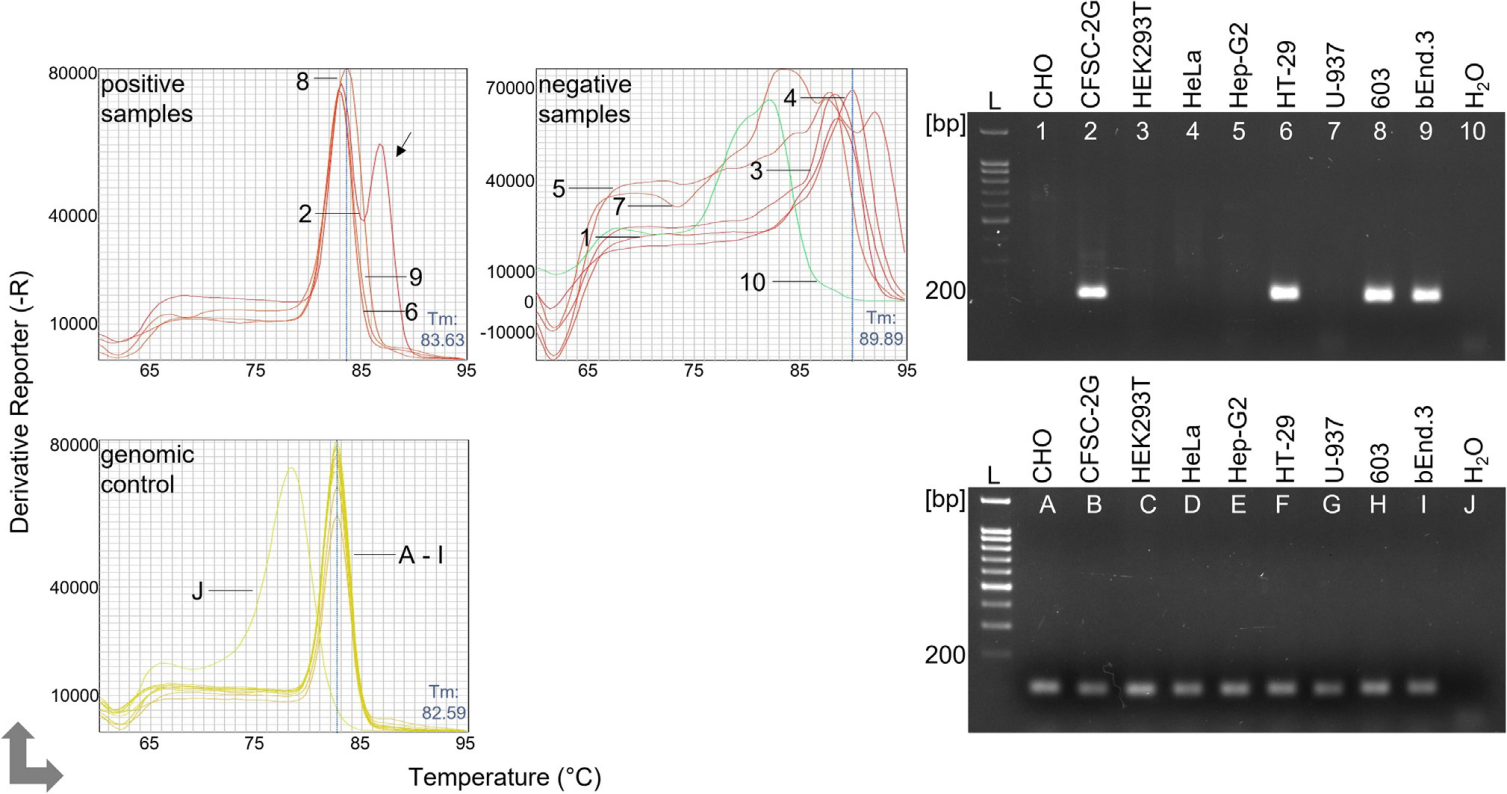
Detection of mycoplasma infection in different cell lines using qPCR qPCR testing was performed with the Myco-primer set using the indicated human, mouse, hamster and rat cell lines. Specific (top left) as well as non-specific (top right) melting profiles are shown. Melting curves for genomic control DNA were generated using the Uc48-primer set (bottom left). The agarose gel images on the right show the corresponding gel electrophoresis results for mycoplasma detection (above) and control (below) qPCR samples. L = DNA ladder, H_2_O = negative control. Numbers in the melting profiles correspond to numbers in the agarose gel image. The arrow indicates the additional peak recorded with the CFSC-2G sample.

Confirming the presence of eukaryotic DNA and indicating productive PCR cycling, single peak melting curves were recorded with all analyzed samples (lower left qPCR melting profile in Figure 3). The presence of a single eukaryotic control DNA amplification product was also directly confirmed by subjecting control PCR samples to agarose gel electrophoresis (lower right gel in Figure 3). As shown in the upper melting profile on the left in Figure 3, three of the four mycoplasma-positive samples produced a single mycoplasma-specific peak. However, the melting curve profile corresponding to the CFSC-2G sample contained a second, non-specific peak (arrow). The appearance of this extra peak shows a possible real-world result and demonstrates that the qPCR machine detectors can record PCR products that are almost invisible in the corresponding agarose gel. By contrast and shown in the upper right melting curve profile, the non-infected cell lines failed to produce the mycoplasma-specific peak but generated non-specific melting profiles (negative samples). In line with this result, gel electrophoresis of mycoplasma-infected qPCR samples confirmed the presence of a mycoplasma-specific amplification product for CFSC-2G, HT-29, 603 and bEnd.3 cells and demonstrated the lack of mycoplasma DNA in all other samples (upper right agarose gel in Figure 3). The corresponding amplification curves of this qPCR experiment can be seen in Figure S2 and document that all mycoplasma-infected samples produced amplification signals earlier than the mycoplasma-negative samples. Because SYBR green qPCR amplification curves do not inform about the specificity of the amplified product, it is not advisable to use Ct cut-off values for deciding whether a sample is mycoplasma-infected or negative. For this reason, Ct values falling below a certain threshold should only be considered as a first indication of mycoplasma infection, and in any case, melting curves and/or agarose gels should be analyzed for a reliable interpretation. In summary, these results show that paired two-primer SYBR green qPCRs are highly suitable for the detection of mycoplasma infections by melting curve analysis and, if indicated, by agarose gel electrophoresis.

### qPCR sensitivity

**Figure 4:**
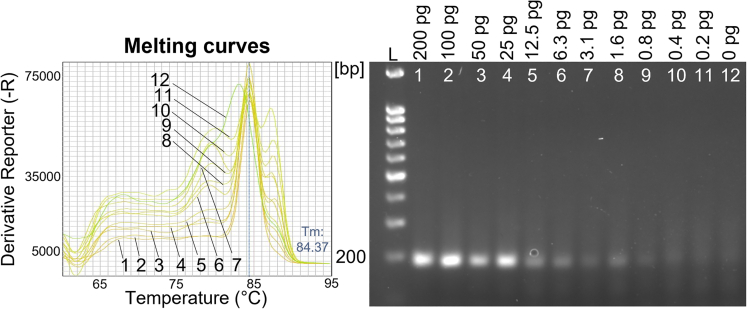
Sensitivity of the two-primer mycoplasma qPCR Melting curve profiles for individual *M. orale* DNA qPCR reactions (left) are shown together with the corresponding agarose gel analysis (right). For each reaction, *M. orale* DNA was serially diluted and mixed with genomic DNA extracted from 1x10^5^ 3T3 cells. *M. orale* DNA concentrations between 200 pg and 0.2 pg were tested. L = DNA ladder.

To assess the limit of detection (LOD) for mycoplasma DNA using qPCR, genomic *M. orale* DNA was serially diluted and added to DNA extracted from 1x10^5^ 3T3 cells followed by a two-primer mycoplasma-specific qPCR. The results indicated a LOD of 0.8 pg or 1.04x10^3^ genomic copies for *M. orale* DNA^31^ in both the melting curve analysis and by using agarose gel electrophoresis (Figure 4). Of interest, we also noted minimal melting temperature (Tm) differences between the individual mycoplasma profiles obtained with *M. orale* DNA and those produced from the four different mycoplasma-infected cell lines (Figure S3). These Tm differences document the amplification of somewhat different PCR products derived from different mycoplasma species, providing further experimental evidence that the universal Myco-primer set is suitable for the detection of different mycoplasma species. Taken together, these results indicate that mycoplasma DNA can be reliably and effectively detected in cell lines at a LOD of 6.3 pg in standard PCR and 0.8 pg when performing qPCR.

### Level of contamination of the positive-tested cell lines

**Figure 5:**
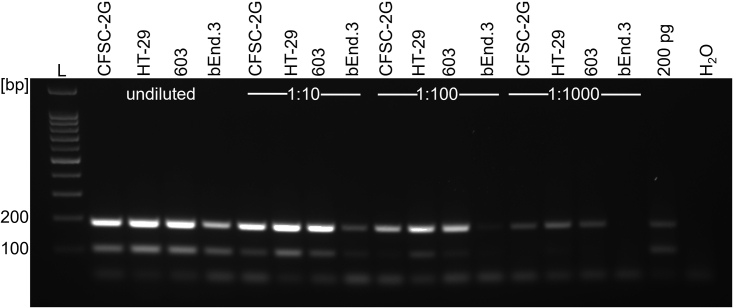
Level of mycoplasma contamination of four infected cell lines by PCR DNA extracts from CFSC-2G, HT-29, 603 and bEnd.3 cell samples were serially diluted (1:10, 1:100, 1:1000) and used for subsequent four-primer PCR testing. As a reference, 200 pg *M. orale* DNA mixed with genomic DNA extracted from 1x10^6^ HEK293T cells was used. The lower 105 bp band represents the internal control PCR product and the upper band (between 166 and 191 bp) indicates the presence of mycoplasma DNA. L = DNA ladder, H_2_O = negative control.

To quantitatively characterize the level of contamination for the mycoplasma-infected cell lines, cell extracts from CFSC-2G, HT-29, 603 and bEnd.3 were serially diluted and subjected to four-primer PCR. As reference, 200 pg *M. orale* DNA mixed with HEK293T DNA was used (Figure 5).

The result of this experiment demonstrated that 1:1000 diluted DNA extracts from the CFSC-2G, HT-29 and 603 cell lines produced comparable amounts of PCR product to 200 pg *M. orale* DNA. For the bEnd.3 cell sample, only the 1:10 dilution gave rise to a clearly detectable mycoplasma-specific band that was comparable to the signal produced by 200 pg *M. orale* DNA. Of note here is that the 1:100 dilution of the bEnd.3 DNA preparation showed no detectable internal eukaryotic control signal, and that the 1:1000 dilution PCR with CFSC-2G, HT-29 and 603 samples produced no internal control PCR product, but clearly generated mycoplasma-specific amplification products. As shown in Figure S4, the two-primer qPCR directly confirmed the results obtained with the four-primer PCR.

In conclusion and with regard to the established LOD, the undiluted samples contained 310 (bEnd.3) and 31.000 (CFSC-2G, HT-29, 603) times more mycoplasma DNA than needed for producing a clear mycoplasma-specific signal in the four-primer PCR protocol and 2500 (bEnd.3) and 250.000 (CFSC-2G, HT-29, 603) times more mycoplasma DNA than needed when using the qPCR protocol.

## Discussion

Historically, mycoplasma detection was tested by microbiological growth on agar and broth or by staining with the Hoechst DNA dye. Now, laboratories use many different commercial and in-house mycoplasma detection methods, but a generally accepted standard has yet to be established. To close this gap, we developed a PCR toolkit that will be useful as a universal standard for routine mycoplasma testing. Because this kit is devised to amplify uniquely conserved 16S rRNA mycoplasma regions together with ultra-conserved eukaryotic DNA sequences, 92% of all known mycoplasma species or strains (not considering duplicate entries within the dataset) are detected and all mammalian and many non-mammalian cell types can be screened.

To the best of our knowledge, this is the first publication showing a detailed listing of all mycoplasma strains that are detected, or conversely, are not covered by the selected primer set, as defined by our *in silico* analysis (Table S1). Bioinformatics analysis also revealed that the optimized mycoplasma primer set detects 92% (226/246) of all mycoplasma strains that are entered in the database. However, when potential matches are considered that do not specify the identity of all nucleotides within the primer binding sequences, these would increase the overall detection coverage to 93% (229/246). The selected mycoplasma-specific primers do not only detect 226 on-target strains but also amplify seven additional off-target strains. These non-mycoplasma matches are species of the genera *Brachyspira*, *Micrococcoides*, *Nitriliruptor*, *Oligella*, and *Peptococcus*. Because the species *Brachyspira hyodysenteriae*, *Peptococcus niger* and *Peptococcus simiae* are anaerobic,^32^^,^^33^^,^^34^ they are absent from standard tissue cultures. The remaining cross-reactive aerobic species are the Gram-positive *Micrococcoides hystricis* and *Nitriliruptor alkaliphilus* and the Gram-negative *Oligella ureolytica*,^35^^,^^36^^,^^37^ all of which have not been reported as cell culture contaminants. Considering that antibiotics normally added to tissue culture media restrict the growth of Gram-positive bacteria and taking into account that unrestricted bacterial growth is evident by the appearance of turbid medium, we are confident that the potential cross-species signals, generated by the above non-mycoplasma species, do not pose a serious threat to the usefulness of our protocol.

As an internal control for functional DNA extraction and PCR cycling, we designed primers targeting highly conserved areas in eukaryotic genomes.^38^ We demonstrate the applicability of these primers for human, mouse, hamster and rat cell lines. In this regard, it is important to note that the targeted sequences are conserved not only in mammals but also in non-mammals including many avian and reptile species. To evaluate the applicability of our toolkit for a specific species, we recommend the UCSC In-Silico PCR tool.^39^^,^^40^

An important consideration in designing a standard testing method was to avoid the addition of exogenous plasmid DNA, which is frequently included in mycoplasma detection protocols.^41^^,^^42^ The advantage of positive control Uc48-primers instead of adding external DNA is that the control PCR signal not only reports a productive PCR cycling, but also demonstrates the presence of a sufficient amount of clean sample DNA. In addition, the absence of external plasmid DNA in the reaction also precludes false positives because of unwanted plasmid carryover and reduces the potential problems of false negatives because of competitive binding to common target sequences present in samples with very low ratios of mycoplasma to plasmid DNA.

Because qPCR is a routine screening method in many laboratories, we tested the practicality of our approach for SYBR green qPCR applications. Given the very similar size of the two amplification products and the resulting complexity of the melting curve profiles, we adapted the original four-primer assay to a paired protocol using only one primer pair in each parallel reaction. As expected, using the paired primer protocol equally resulted in highly reliable qPCR readouts represented by melting curve analysis or agarose gel electrophoresis. Nevertheless, for routine laboratory screening, we recommend the four-primer setup because it is simple, reduces sample preparation times and is more cost-effective.

One additional consideration for mycoplasma detection is sensitivity. To determine the sensitivity of the method, we utilized serial dilutions of *M. orale* DNA*.* To faithfully reflect the conditions of the standardized detection protocol, equal amounts of cell line DNA were added to each PCR sample. We intentionally used *M. orale* DNA for sensitivity testing because this mycoplasma strain has a very low percentage of genomic copies per colony-forming unit (GC/CFU), thus representing a detection target with an overall low genomic mycoplasma DNA content.^31^ Although our detection limits of 6.3 pg for the standard PCR and 0.8 pg mycoplasma DNA for the qPCR protocol are above those reported by some commercial kits or other protocols,^43^^,^^44^^,^^45^ the here presented method will be more than sufficient for reliable routine laboratory testing, as shown in Figures 5 and S4. This result is not surprising, considering that a single mycoplasma bacterium forms about 10^6^ CFU/mL within 3–5 days^6^ and taking into account that contaminated cell cultures usually contain about 10^6^-10^8^ CFU/mL.^6^^,^^10^ Therefore, four-primer PCR testing will definitely detect mycoplasma contamination in cultures growing longer than three days and also in cryopreserved aliquots. A second argument for the reliability of our PCR method in terms of mycoplasma testing is the use of DNA cell extracts. Because mycoplasmas grow attached to cell membranes^9^ or replicate within the cytoplasm,^6^^,^^7^ each infected cell contains on average 100–1000 individual mycoplasma cells.^6^ The use of DNA cell extracts instead of culture supernatants in our toolkit therefore massively enriches mycoplasma DNA and thus highly increases the number of starting templates.

To isolate DNA from cell pellets, we intentionally devised a protocol that enables simple, reliable and inexpensive DNA isolation, which is useful for routine mycoplasma screening. If sensitivity is critical, it will be possible to increase the overall detection limit by using commercial DNA isolation kits that minimize potentially sensitivity-limiting substances such as SDS, EDTA (components in our lysis buffer) or proteins.^46^ In addition, detection limits can also be further increased by fine-tuning measures, e.g., by touchdown PCR,^47^ by utilizing thiol-modified primers^48^ or by using different polymerases. A good example, where a very high PCR sensitivity is required, is the screening of cell therapy products for medical use. For reaching the minimum LOD of 10 CFU in such applications, specialized commercial kits^43^^,^^44^ or TaqMan-based multi-primer qPCRs^45^ are needed. However, the here presented mycoplasma detection system is not intended for specialized applications but designed as a generally applicable and highly reliable standard for routine mycoplasma testing.

The great diversity of current mycoplasma detection methods and the different compositions of the individual PCR primer sets used prevent a direct comparison of results and lead to questionable heterogeneity of results. We would therefore argue that a standardized method for regular testing removes this bias and introduces objective standard measurements. The PCR method presented here meets all the expected requirements for mycoplasma testing, such as good specificity, consistent sensitivity, high coverage at low cost and is therefore a very suitable standard for regular mycoplasma testing.

### Limitations of the study

The presented method is optimized for routine cell culture testing and provides also highly reproducible results for cryopreserved cell stocks. Although the functionality of the ultra-conserved mycoplasma-specific primers has been confirmed with *M. orale* DNA and four mycoplasma-infected cell lines, all different 198 mycoplasma species matching both targeted sequences have not been formally tested. However, considering the high reliability standards of public databases and taking into account the PCR amplification mechanism, the selected Myco-primer set is expected to recognize all matched mycoplasma-specific sequences and thus cover most, if not all, genus- and strain-specific on-target entries listed in Tables 1 and S1. A second caveat concerns the correct interpretation of the qPCR reactions. Because SYBR green qPCR amplification curves do not indicate the specificity of the amplified product, Ct values falling below a predefined threshold should be considered only as a first indication of mycoplasma infection and must be confirmed by melting profiles and/or agarose gel electrophoresis. In addition, because the protocol detects many different mycoplasma species that generate different PCR products (166–191 bp), species-specific amplicons with variable secondary structures may result in altered amplification efficiency that can affect individual Ct values.^49^ For this reason, Ct values do not allow proper quantification or comparison of contamination levels between cell lines that may be infected with different mycoplasma species. The method is not suitable for testing of medical GMP cell products intended for human use. If ultra-high detection sensitivity is needed, the provided primers will be the optimal primer combination, but it might be necessary to improve mycoplasma DNA extraction and PCR cycling. Finally, the method will miss seven to 8% of mycoplasma species and if all known mycoplasma strains are to be covered, additional primer sets must be used.

## STAR★Methods

### Key resources table

**Table:** 

REAGENT or RESOURCE	SOURCE	IDENTIFIER
**Biological samples**		

*Metamycoplasma orale* DNA	Deutsche Sammlung von Mikroorganismen und Zellkulturen	Cat# DSM 25590

**Critical commercial assays**		

5x ready to load PCR master mix	Bio&SELL	Cat# BS91.731.1000
Luna® Universal One-Step RT-qPCR Kit	New England Biolabs	Cat# E3005E

**Deposited data**		

Bacterial 16S Ribosomal RNA	NCBI Refseq Targeted Loci Project	PRJNA33175

**Experimental models: Cell lines**		

bEnd.3	ATCC	Cat# CRL-2299, RRID:CVCL_0170
CFSC-2G	Prof. M. Rojkind	Greenwel et al.,^50^^,^^51^ RRID:CVCL_4U34
CHO	ATCC	N/A, RRID:CVCL_0213
D122-84	stored in lab	Eisenbach et al.^52^
HCmel12	stored in lab	Bald et al.^53^
HEK293T	ATCC	Cat# CRL-3216, RRID:CVCL_0063
HeLa	ATCC	Cat# CCL-2, RRID:CVCL_0030
Hep-G2	ATCC	Cat# HB-8065, RRID:CVCL_0027
HT-29	ATCC	Cat# HTB-38, RRID:CVCL_0320
JAWSII	ATCC	Cat# CRL-11904, RRID:CVCL_3727
LuCa6	generated in lab	Rosigkeit et al.^54^
MC38	Abm	Cat# T8291
MutuDC1	Prof. H. Acha-Orbea	Fuertes Marraco et al.^55^
U-937	ATCC	Cat# CRL-1593.2, RRID:CVCL_0007
3T3	ATCC	Cat# CRL-1658, RRID:CVCL_0594
603	stored in lab	Versteeg et al.,^56^ RRID:CVCL_UM78

**Oligonucleotides**		

Myco forward: TYC TAC GGG AGG CAG CAG	This paper	N/A
Myco reverse: CGR CTG CTG GCA CAT AGT T	This paper	N/A
Uc48 forward: TGT CCT GGA GTT TGG CTT GG	This paper	N/A
Uc48 reverse: TAG TAG CAG CCT AGC ACC CA	This paper	N/A

**Software and algorithms**		

MAFFT (Multiple alignment using fast Fourier transform)	Katoh et al.^57^	https://www.ebi.ac.uk/Tools/msa/mafft/, RRID:SCR_011811
ClustalX2 V5	Madeira et al.^39^	http://www.clustal.org/clustal2/, RRID:SCR_017055
Microsoft Excel	Microsoft	N/A
UCSC In-Silico PCR	Kent et al.^58^	https://genome.ucsc.edu/cgi-bin/hgPcr, RRID:SCR_003089
Primer-BLAST	Ye et al.^59^	https://www.ncbi.nlm.nih.gov/tools/primer-blast/, RRID:SCR_003095

### Resource availability

#### Lead contact

Further information and requests for resources and reagents should be directed to and will be fulfilled by the lead contact, Dominik Siegl (domsiegl@uni-mainz.de).

#### Materials availability

This study did not generate new unique reagents except for the in-here published primer sequences.

### Experimental model and subject details

#### Cell lines

16 different cell lines derived from human, rat, mouse or hamster were used for DNA extraction. bEnd.3 (sex unknown), CHO (female), HEK293T (female), HeLa (female), Hep-G2 (male), JAWSII (sex unknown), U-937 (male) and 3T3 (sex unknown) were originally provided by the American Type Culture Collection (ATCC), MC38 (female) was obtained from Abm. The LuCa6 (sex unknown) cell line was in-house generated by isolating tumor cells of the CC10-CreERT2 Kras^LSLG12Vgeo/WT^ Trp53^fl/fl^ C57BL/6 mouse model.^54^ Cell lines D122-84 (sex unknown),^52^ HCmel12 (sex unknown)^53^ and 603 (sex unknown)^56^ are referenced. CFSC-2G,^50^^,^^51^ and MutuDC1^55^ cell lines were kindly provided by Prof. M. Rojkind and Prof. H. Acha-Orbea, respectively.

CHO, HEK293T, HeLa LuCa6, MC38, U-937 and 3T3 cell lines were cultured in DMEM-Glutamax (Gibco, Thermo Fisher Scientific) supplemented with 10% fetal bovine serum (FBS), 100 U/ml penicillin and 100 μg/ml streptomycin (Gibco, Thermo Fisher Scientific). JAWSII were cultured in IMDM-Glutamax (Gibco, Thermo Fisher Scientific) supplemented with 10% FBS, 100 U/ml penicillin,100 μg/ml streptomycin and 5 ng/ml GM-CSF. Cells were propagated using standard tissue culture parameters (37°C humidified, 5% CO_2_). CFSC-2G, Hep-G2, HT-29, 603, bEnd.3, D122-84, HCmel12 and MutuDC1 cell lines were not propagated, and DNA extraction was performed using a cryopreserved aliquot.

### Method details

#### Identification of ultra-conserved primers

##### Mycoplasma primers

The 16S rRNA sequences of 60 mycoplasma strains (including *Acholeplasma laidlawii*) were obtained from the NCBI Bacterial 16S Ribosomal RNA RefSeq Targeted Loci Project (Accession PRJNA33175). These sequences were aligned using the tool MAFFT (**M**ultiple **a**lignment using **f**ast **F**ourier **t**ransform)^57^ and imported in ClustalX2^39^ for analysis. Highly conserved areas shared by all 60 analyzed strains were selected and a single degenerative nucleotide was introduced in each primer to directly match all sixty DNA sequences. To characterize all strains and species detected by the defined primer pair, the complete dataset consisting at that time (January 3^rd^, 2023) of 25796 sequences (Accession code PRJNA33175) was imported into Excel. To exclude potential cross-reacting primer matches, we used the UCSC In-Silico PCR tool with standard parameters on human (Assembly Dec. 2013 (GRCh38/hg38)) and mouse (Assembly Dec. 2011 (GRCm38/mm38)) genomic databases.^40^^,^^58^ The designed primer pair (synthesized by Metabion international AG, Germany) for mycoplasma detection (Myco-primers) is listed in the key resources table and generates a 166-191 bp product, which depends on the amplified mycoplasma strain.

##### Internal control primers

To identify suitable control primers serving as internal positive PCR controls, ultra-conserved (Uc) human genomic elements^38^ were subjected to NCBI primer-BLAST using a melting temperature of 60°C ± 3°C.^59^ These ultraconserved elements were chosen since one criterion for qualifying the mapped elements as potential PCR targets was that their sequences should be identical to the genomic sequences of commonly used mammalian cell lines such as human, mouse, monkey, rat, and Chinese hamster, and that the target sequences should also cover a range of avian (e.g. chicken) and reptile species. The designed primer pair (synthesized by Metabion international AG, Germany) for amplifying an ultra-conserved eukaryotic sequence (Uc48-primers) is listed in the key resources table and generates a 105 bp product.

#### DNA extraction

An aliquot of cells (cryopreserved aliquot or directly harvested) was used containing at least 100.000 cells (in PBS, FCS, culture- or freezing medium). The suspension was centrifuged (12000 g/2 min) and the supernatant discarded. Subsequently, cells were digested in 40 μl lysis buffer (100 mM TRIS pH 8.5, 5 mM EDTA, 0.2% SDS, 200 mM NaCl) supplemented with 10 μl proteinase K (10 mg/ml in ddH_2_O) for at least 4h at 56°C or overnight at 37°C. Prior to the PCR, 150 μl of autoclaved ddH_2_O were added, the mixture incubated at 95°C for 5 min and centrifuged to pellet cellular debris. The supernatant containing the DNA can be stored at 4°C for several weeks or at -20°C for long-time storage.

#### PCR

The PCR conditions were optimized using the Bio&SELL “5x ready to load” master mix (BS91.731.1000, Bio&SELL GmbH, Germany) in a total volume of 25 μl. 2 μl of the extracted DNA solution were used together with a final concentration of 0.6 μM of each mycoplasma detection primer and 0.4 μM of each internal control Uc48-primer. Cycling: 95°C/3 min (initial denaturation) followed by 40 cycles of: 95°C/30 sec, 64°C/30 sec, 72°C/40 sec and a final extension at 72°C for 5 min (BioRad iCycler). 12 μl of the completed PCR samples as well as 5 μl of the 100 bp plus DNA marker (BS96.329.0050, Bio&SELL GmbH, Germany) were loaded on a 2% agarose gel containing 2 drops of a 0.025% ethidium bromide solution (Carl Roth GmbH & Co. KG, Germany) and the amplified PCR fragments were visualized using the ChemiDoc XRS+ gel imaging system (Bio-Rad Laboratories, Inc., USA).

#### Quantitative PCR (qPCR)

qPCRs were performed using the Luna® Universal One-Step RT-qPCR Kit (E3005E, New England Biolabs, USA) with 2 μl extracted DNA solution in a total volume of 20 μl. In the positive control reaction, a final concentration of 0.4 μM of each control Uc48-primer was used and in the mycoplasma detection reaction, a final concentration of 0.6 μM for each Myco-primer were used. Cycling: 95°C/1 min (initial denaturation) followed by 40 cycles of: 95°C/15 sec, 64°C/1 min. Melting curve: 60-95°C, ramp +0.3°C in 15 second steps (Applied Biosystems, StepOnePlus Real-Time PCR system). To visualize the products (ChemiDoc XRS+, Bio-Rad Laboratories Inc., USA), the completed PCR samples were mixed with 10x loading buffer (200 mg Bromphenol blue, 50 mg Xylene cyanol, 20 ml 0.5 M EDTA (pH 8), 90 ml Glycerol filled up to 120 ml with ddH_2_O) and 12 μl were loaded on a 2% agarose gel containing 2 drops of a 0.025% ethidium bromide solution (Carl Roth GmbH & Co. KG, Germany). 5 μl of the 100 bp plus DNA marker were applied to the same gel (BS96.329.0050, Bio&SELL GmbH, Germany).

#### Limit of detection (LOD) assay

To evaluate the LOD with a characterized strain and using precisely defined amounts of starting material, an aliquot of *Metamycoplasma orale* DNA was purchased (DSM 25590, Deutsche Sammlung von Mikroorganismen und Zellkulturen GmbH, primary depositor K. Yamamoto). For PCR, serial 1:2 dilutions starting with a total amount of 20 ng/150 μl were prepared and added to 50 μl of DNA extracted from 1x10^6^ HEK293T cells. For qPCRs, serial 1:2 dilutions starting with a total amount of 20 ng/150 μl were prepared and added to 50 μl of DNA extracted from 1x10^5^ 3T3 cells. PCR and qPCR were performed as described.

#### Level of contamination assay

To quantify and characterize the level of mycoplasma DNA in the contaminated CFSC-2G, HT-29, 603 and bEnd.3 cells, serial 1:10 DNA dilutions were prepared (1:10, 1:100, 1:1000). As reference, 200 pg *M.* *orale* DNA mixed with DNA extracted from 1x10^6^ HEK293T cells was used. PCR was performed as described.

## Data Availability

• This paper analyzes existing, publicly available data. The accession number for the dataset is listed in the key resources table and individual accession numbers are shown in Table S1. • This paper does not report original code. • Any additional information required to reanalyze the data reported in this paper is available from the lead contact upon request.

## Supplemental information

Document S1. Figures S1–S4

Table S1. All on-target mycoplasma matches, potential mycoplasma matches, missing mycoplasma matches and all off-target matches that were extracted from the NCBI Bacterial 16S Ribosomal RNA RefSeq Targeted Loci Project (Accession PRJNA33175) are listed, related to Table 1
