# Supplementary material for: A PCR protocol to establish standards for routine mycoplasma testing that by design detects over ninety percent of all known mycoplasma species
Source: iScience. 2023 Apr 26;26(5):106724. doi: 10.1016/j.isci.2023.106724 (PMC10192841; doi:10.1016/j.isci.2023.106724)
Supplement: Document S1. Figures S1–S4 [file mmc1.pdf]

iScience, Volume *26*

Supplemental information

A PCR protocol to establish standards for routine mycoplasma testing that by design detects over ninety percent of all known mycoplasma species

Dominik Siegl, Marie Kruchem, Sandrine Jansky, Emma Eichler, Dorothe Thies, Udo Hartwig, Detlef Schuppan, and Ernesto Bockamp

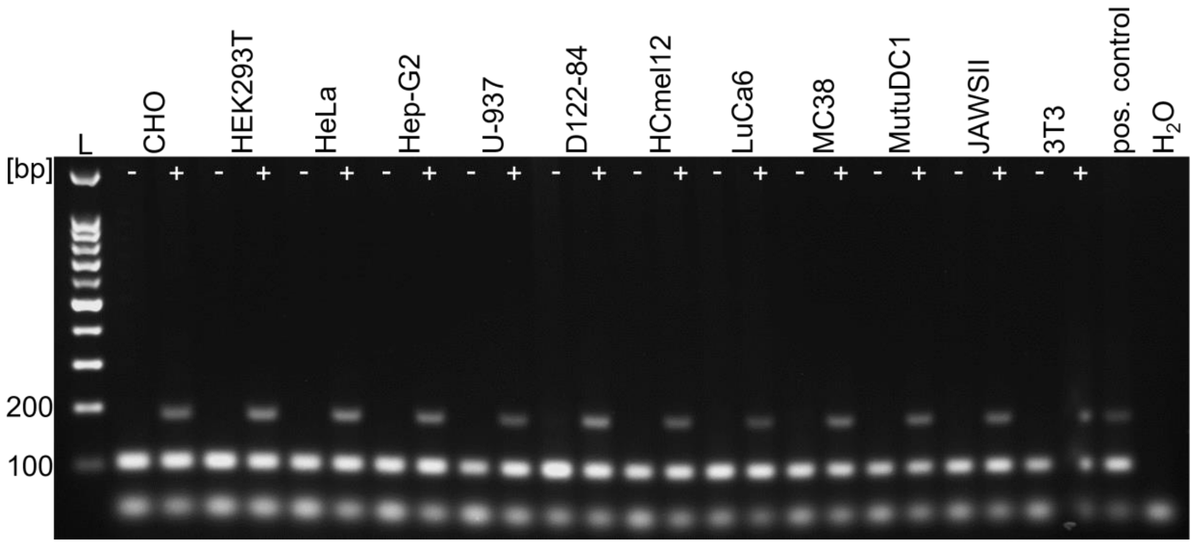

Figure S1 Four-primer PCR produces a mycoplasma-specific signal in the context of genomic DNA extracted from different eukaryotic cell lines. Four-primer PCR was performed using the indicated twelve cell lines of human, mouse and hamster origin. PCR was performed with mycoplasma-negative eukaryotic DNA samples (-) and in parallel with identical amounts of mycoplasma-negative eukaryotic DNA that was mixed with 50 pg *M. orale* DNA (+). The lower 105 bp band represents the internal control PCR product and the upper 188 bp band indicates the presence *of M. orale* DNA. L = DNA ladder, pos. control = DNA extracted from mycoplasma-infected cells, H_2O = negative control. Related to Figure 2.

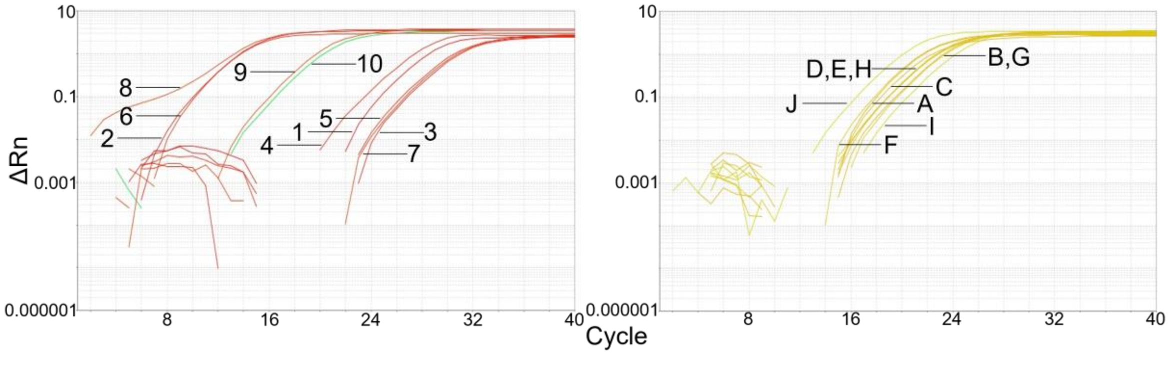

Figure S2 Amplification curves of mycoplasma-infected and mycoplasma-negative cell lines generated by qPCR. The left plot depicts amplification curves generated with the Myco-primer set documenting that all mycoplasma-infected samples (labeled 2, 6, 8 and 9) produce detectable qPCR amplification signals earlier than mycoplasma-negative samples (labelled 1, 3, 4, 5, and 7). The right plot depicts amplification curves generated with the host cell-specific Uc48-primer set showing mycoplasma-infected samples (labelled B, F, H and I) and mycoplasma-negative samples (labelled A, C, D, E and G). Sample numbers and letters correspond to the numbers/letters shown in the agarose gel images of related Figure 3.

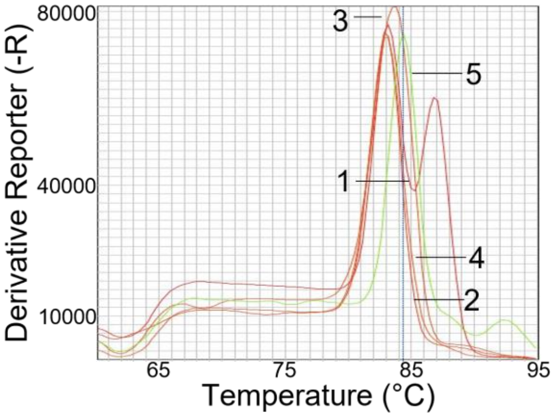

Number	Origin/Sample	Melting temperature [Tm]
1	CFSC-2G	83.19
2	HT-29	83.03
3	603	83.63
4	bEnd.3	83.04
5	*M. orale*	84.38

Figure S3 Individual melting curve profiles of four mycoplasma-infected samples and *M. orale* DNA. All qPCR reactions were performed with the Myco-primer set. Individual melting temperatures (Tms) for each qPCR reaction are shown on the right, indicating the presence of different mycoplasma strains. Related to Figure 3 and Figure 4.

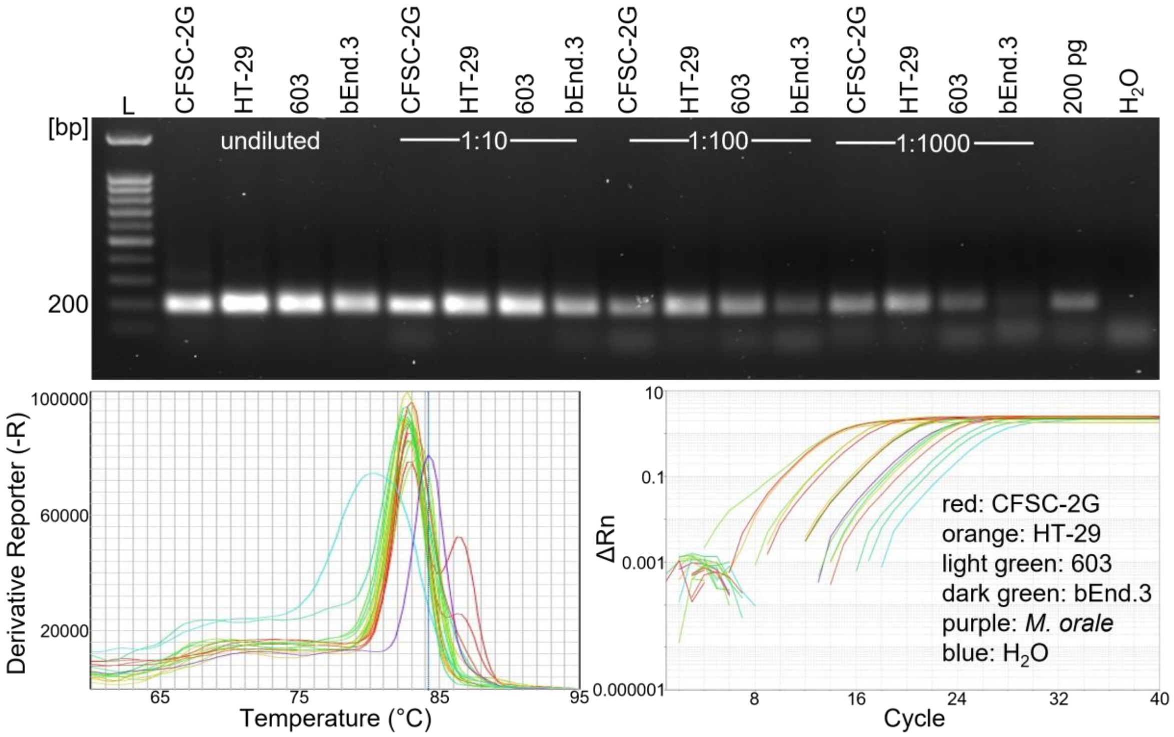

Figure S4 Level of mycoplasma contamination of four infected cell lines by qPCR. DNA extracts from CFSC-2G, HT-29, 603 and bEnd.3 cell samples were serially diluted (1:10, 1:100, 1:1000) and used for subsequent two-primer qPCR testing. As a reference, 200 pg *M. orale* DNA mixed with genomic DNA extracted from $1x10^6$ HEK293T cells was used. The corresponding agarose gel electrophoresis result (top), the individual melting profiles (bottom left) and amplification curves (bottom right) are shown. L = DNA ladder, H_2O = negative control. Related to Figure 5.
